# Factors influencing ease of whelping and its relationship with maternal behaviour and puppy perinatal mortality in commercially bred dogs

**DOI:** 10.1038/s41598-022-10707-w

**Published:** 2022-04-23

**Authors:** Uri Baqueiro-Espinosa, Victoria McEvoy, Gareth Arnott

**Affiliations:** Institute for Global Food Security, School of Biological Sciences, Queen’s University, Belfast, UK

**Keywords:** Animal behaviour, Animal physiology

## Abstract

For dog breeders, parturition is a critical stage in the reproductive cycle of the dam. Evidence in other mammals suggests that a difficult labour can influence maternal behaviour and offspring viability during the first hours postpartum. However, the effect of whelping difficulty on the onset of maternal behaviour has not yet been investigated in domestic dogs. Here we developed an ease of whelping (EoW) index in dams maintained within a Commercial dog Breeding Establishment (CBE) environment and investigated the relationship between intrinsic and extrinsic factors (breed group according to size/weight, litter size, parity, whelping season and origin of the dam), EoW, early maternal behaviour and puppy perinatal mortality. The behaviour of 30 dams was observed throughout the whelping process, starting 24 h before delivery of the first puppy until birth of the last puppy. Parturition duration, birth interval, and behaviours indicative of distress, restlessness, and general activity were scored and included in a Principal Component Analysis to construct the EoW index. Subsequently, mother–pup interactions and puppy perinatal mortality were recorded during the first 24 and 72 h postpartum respectively. Results showed that EoW was significantly affected by whelping season, litter size and origin of the dam (whether she was born and raised within the CBE or brought in). Furthermore, mothers that experienced more difficult parturitions (higher EoW score) spent more time lying in contact with their puppies during the first 24 h postpartum. Time in contact with puppies was also significantly affected by breed group. Nursing duration was significantly affected by breed group and origin of the dam. Additionally, medium-size breed (10–20 kg) puppies were significantly less likely to experience perinatal mortality than large breeds (> 20 kg). These findings are particularly relevant for the welfare of breeding dams maintained in large-scale CBEs where the staff-to-dog ratio might be insufficient to adequately manage multiple simultaneous parturitions.

## Introduction

The rising popularity of dogs as companion animals has led to the emergence of commercial breeding operations, referred to in the literature as Commercial Breeding Establishments (CBEs), which supply the high demand for purebred and designer crossbred dogs in developed countries^1^. Regulations for dog breeders vary between countries^2^, thus considerable differences exist in management practices and dog living conditions across CBEs. Facilities range from breeders producing only a few litters per year from limited breeding stock to those producing hundreds of litters from hundreds of breeding animals^3^. Although large-scale breeders are often stricter about prevention and management of disease within their breeding facilities, they also tend to provide a less enriched environment compared to smaller breeders^3^. Dogs maintained within large-scale breeding facilities usually spend most of their life inside kennels with restricted space and limited or no access to exercise, enrichment or positive human interaction^4^. Previous studies carried out on shelter and laboratory dogs have demonstrated the detrimental effects that confined environments can have on the welfare of dogs, which may become chronically stressed^5–9^. Moreover, a retrospective survey reported that rehomed CBE dogs formerly used as breeding stock were more likely to exhibit stranger-directed and non-social fear related behaviours, anxiety and compulsive behaviours compared with pet dogs obtained from a different source^4^. A possible explanation is that large-scale breeding facilities commonly restrict access for strangers, meaning that dogs have few or no positive interactions at all with unfamiliar people^3^. Additionally, human contact may be reduced even further before parturition, when pregnant dams can be segregated from their conspecifics and housed individually for several weeks with the objective of minimising disturbance^10^.

The periparturient period is considered by breeders as the most important stage during the breeding process^11^ and can be a highly stressful event for both mother and offspring^12^, especially at the onset of labour^13^. In dogs, this can be reflected in the behaviour of the dam up to 24 h before delivery of the first puppy, as shown by the escalation of behavioural signs such as restlessness, nesting behaviour, panting and barking^14^. Periparturient behaviour has been shown to be a reliable indicator of difficulty of parturition in other domestic species such as pigs^15^, dairy cows^16^ and laboratory rats^17^. A difficult or disturbed parturition, known as dystocia, represents a risk for the life of the mother and the newborn puppies^18^. The incidence rate of canine dystocia is highly variable^19,20^, although it has been reported to be as high as 28% among certain populations^21^. Factors such as breed, litter size, parturition duration, and season of whelping have been associated with the occurrence of dystocia and perinatal mortality in dogs^18,22–24^. Small and miniature breeds have a higher risk for dystocia^18^. Cornelius et al.^24^ reported that risk for dystocia also increased with age of the mother and dams with small and large litters (< 5 and > 9 puppies respectively) were more likely to experience a difficult whelping compared with those giving birth to medium-sized litters. Similarly, the risk of stillbirth is higher for large litters (> 11 puppies), puppies with longer interbirth intervals^24^, and positively correlated with duration of expulsion^18^.

In addition to the above mentioned factors, a stressful environment or event can have repercussions on the birthing process^12^. Increased cortisol levels have been observed in cows experiencing dystocia^25^ and in late pregnant cows after being transported by road, a well-documented stressful event for farm animals^26^. Likewise, transport increased cortisol concentration and accelerated the onset of foaling in mares^27^. On the other hand, the expulsive phase was prolonged, increasing foaling duration, in pony mares moved to an unfamiliar and less comfortable environment right at the onset of parturition^28^. Similar phenomena have been observed in polytocous species where external stressors may interrupt and delay parturition by prolonging the birth interval between two consecutive littermates^29–31^. In pigs, a species often compared with dogs regarding their cognitive and interspecific communicative abilities^32^, Andersen et al.^33^ reported that sows showing higher fear reactions towards humans had longer farrowing durations and spent less time resting before the onset of farrowing. Moreover, fearful or reactive sows also experienced prolonged inter-birth intervals^34^. Chronic mild stress during gestation has been found to impair maternal behaviour in female mice (*Mus musculus*), affecting their ability to protect their offspring against an intruder^35^ and reducing nursing and grooming rates and the amount of time spent in the nest^36^. Research examining the effect of environmental stress on the whelping process in dogs is scarce^12^. Similar to other species, the delivery of the first puppy can be delayed by the dam being anxious due to an unfamiliar or stressful environment^20,37^. Based on the evidence found in other species, it can be hypothesised that breeding dams maintained under analogous intensified living conditions may suffer from similar effects during parturition as a result of a stressful environment.

The amount and quality of maternal care can affect the offspring’s temperament as adult dogs^38^. More importantly, maternal behaviour plays an essential role in puppy survival during the first hours postpartum. Shortly after delivery of each puppy, the mother breaks the foetal membranes and licks the newborn’s head and mouth, stimulating respiration^39,40^. The absence of or delay in these behaviours can lead to the puppy’s death due to asphyxiation in the sac or as a consequence of hypoxia during the first hours postpartum^41,42^. Other behaviours such as lying in contact with the puppies, licking a puppy’s head and anogenital area and nursing are also essential for offspring survival during the first hours and days postpartum^40^. These behaviours help to maintain the puppy’s body temperature^43^, stimulate the excretion process^44^ and facilitate the ingestion of colostrum^40^. Previous studies have investigated the effect of different extrinsic and intrinsic factors on the quality of maternal behaviour in dogs, including breed, parity, litter size, and whelping season^38,45,46^. However, the relationship between whelping difficulty and maternal behaviour during the first hours postpartum has not yet been explored. Evidence in other domestic species shows that difficulty of parturition can affect the mother’s behaviour shortly after delivery. Cows that experienced difficult calvings, and thus required human assistance, spent more time laying down in a lateral position with the head rested^16^. In sows, latency to leave the farrowing hut decreased after longer farrowings^47^. Duration of labour and the amount of assistance received has also been shown to affect the latency to groom the first lamb in ewes^48^. Furthermore, ewes that had extended and difficult lambings spent less time licking their lambs and were more likely to reject them or move away when they attempted to suck^49^. These results suggest that longer and more difficult parturition may be associated with exhaustion, pain and discomfort in the dam during the early postpartum period, which in turn might affect maternal behaviour and offspring survival. Moreover, Dos Santos et al.^11^ reported that abnormal and maladjusted maternal behaviours like ignoring the puppies and cannibalism was observed more often in larger dog-breeding kennels.

Generally, only parturitions requiring manual intervention, medical treatment or emergency caesarean section are classified as dystocia in the literature^20^. However, birth difficulty might also vary between eutocic dams with apparently undisturbed parturitions. Furthermore, the high dog-to-staff ratio often found in large-scale breeding facilities reduces the amount of human contact for dogs^11^, and may prevent appropriate supervision of dams around whelping. Therefore, it is important to identify litters that could have suffered difficult parturitions, and which puppies might require additional attention during the first days of life. The aims of the current study were: (1) to develop an ease of whelping (EoW) index based on the behaviour of the dam 24 h before and during parturition, (2) to determine which factors, such as parity, litter size, breed, origin of the dam and whelping season, may influence EoW in CBE dogs, and (3) to investigate the association between EoW, intrinsic and extrinsic factors and maternal behaviour during the first 24 h postpartum and perinatal mortality within 72 h postpartum. We hypothesised that mothers experiencing more difficult whelpings (i.e. higher EoW scores) would show impeded maternal behaviour expressed as fewer mother-puppy interactions and would be more likely to have stillborn and dead puppies during the first 72 h postpartum.

## Materials and methods

### Ethical statement

This study received ethical approval from the Queen’s University Belfast School of Biological Sciences Ethics Committee (approval number: QUB-BS-AREC-19-004). We confirm that all methods were performed in accordance with the relevant guidelines and regulations.

### Subjects, housing and management

Thirty pregnant dams were selected to participate in this study from a fully licensed Commercial dog Breeding Establishment located in the United Kingdom. The dogs varied in breed and all of them were older than one year of age. The parity of the dams ranged from 0 to 4. Twelve dams were born and reared within the CBE and 18 were born elsewhere and brought into the CBE at different ages. As the first objective of the study was to create an EoW index for undisturbed parturitions, only dams which did not require medical intervention or caesarean section were included in the study. The entire information for the 30 dams included in the study can be found in Supplementary Table S1.

Within the CBE, dogs were normally housed in kennels in groups of two to four females. Approximately one week before the expected date of parturition (63 days after first mating), pregnant dams were moved into individual whelping pens located in an enclosed building, where they were kept until the weaning process started around six weeks after giving birth. This area was equipped with a heating system which was operated at the breeder’s discretion. Whelping pens were made of painted rectangular corrugated metal sheets and varied in size from 3 m length by 1 m width by 1 m height to 3 m length by 1.10 m width by 1.28 m height. This design prevented dogs from seeing the adjacent pen but allowed them to smell each other through the gap between the lateral panel and the floor. Each pen contained either a small or a large wooden whelping box located at the back, its size depending on the size of the kennel (small box: 60 cm by 60 cm by 60 cm; large box: 90 cm by 110 cm by 96 cm). All whelping boxes had an entrance cut either on the side or front panel which allowed the mother free access in and out of the box. Heating lamps were hanged by the breeders above the whelping boxes to help regulate the puppies’ temperature usually before the expected date of parturition and throughout the first days postpartum. The floor of the whelping pens was made of concrete and covered in sawdust, which was partially changed at least once a day. Dogs were provided with straw as bedding material inside the whelping boxes. Water and dry food (Royal Canin Professional Mini Starter, Crown Pet Foods Ltd., Somerset, UK) were offered ad libitum throughout the day. Additionally, breeding dams were supplemented once a day with a high-calorie meal consisting of wet cat food (Tesco cuts in jelly with tuna, Tesco Stores Ltd., Cheshunt, UK) and ewe’s milk (Novilam, Schils BV, Sittard, The Netherlands) mixed with dog kibble. Kennels were cleaned twice a day, around 8:00 am and 5:00 pm.

### Data collection

The study was performed from September 2019 to September 2020. Surveillance cameras (Swann SWPRO-1080MSBPK2-UK) were used to record the behaviour of the dogs throughout the duration of the study. Before each dam was moved into a whelping pen, two cameras were placed inside each pen, one at the top of the front of the pen and one inside the whelping box. The behaviour of the dams was continuously recorded on video from the day they were moved into the whelping pens until three days after giving birth. Video files were downloaded remotely from the DVR and stored in a hard drive for later behavioural scoring and analysis.

To investigate which factors might influence ease of whelping and maternal behaviour, breed, parity and origin of the dam (whether she was born and reared in the CBE or born elsewhere and brought into the CBE) were obtained from the breeder’s records. Additionally, litter size, number of stillbirths, number of dead puppies at 72 h postpartum, and season of whelping were recorded for each litter. Whelping seasons were categorised according to the meteorological temperate seasons^50^ as Winter (December–February), Spring (March–May), Summer (June–August) and Autumn (September–November). Dams were classified into three different breed groups according to their breed standard average size/weight: small breeds (< 10 kg), medium-sized breeds (10–20 kg) and large breeds (> 20 kg).

#### Recording procedure for ease of whelping

Before starting data collection, video footage was observed to determine the starting and ending times of whelping for each dam. Parturition prodromal signs and pre-birth behaviours, believed to be triggered by the increase of the hormone prolactin, can start as early as 24 h before delivery of the first puppy^20^. Thus, video footage was used to score the behaviour of each dam for the first 15 min of every hour during the 24 h before the birth of the first puppy (pre-parturition period, 6 h total) and throughout the whole duration of parturition until the birth of the last puppy. Based on the literature, a total of 11 behaviours indicative of activity, restlessness, distress, discomfort and relaxation were included in an ethogram (Table 1) and their duration and frequencies were continuously recorded^14,20,51^. Total duration of whelping (time elapsed in minutes between the births of the first and the last puppy) and birth interval (time in minutes between the births of two consecutive puppies) were also recorded for each litter.

**Table 1:** Behavioural variables recorded for the ease of whelping index and measured as durations (D) or frequencies (F) of occurrence.

Behaviour	Description	Interpretation	Frequency/duration
Standing	Dog is standing with four paws on the floor and legs extended supporting her body	2, 4	D
Sitting	Dog is sitting and supporting the weight of the body on her hind legs	5	D
Lying down	Dog is resting on the floor in sternal, lateral or dorsal recumbency	5	D
Position change	Number of changes between standing, sitting and lying down positions	1, 2, 4	F
Walking	Dog walks around the whelping pen moving to a new position within the enclosure	1, 2	D
Circling	Dog walks in circles inside or outside the whelping box	1, 2, 4	D
Nest building behaviour	Dog uses her forelimbs to scratch the bedding or surface inside the whelping box or nudges bedding with the nose	1, 2	D
Self-directed behaviour	Grooming, scratching, licking behaviours directed towards the dog’s own body	4	D
Elimination behaviour	Dog excretes urine or faecal material	3, 4	F
Retching	Dog attempts to disgorge the stomach contents leading to productive or non-productive vomiting	3, 4	F
Vocalisation	Dog mouth opens and closes producing short or long duration sounds such as barking or howling	3	F

Behaviours were interpreted as being indicative of activity (1), restlessness (2), distress (3), discomfort (4) or relaxation (5)^14,20,51^.

#### Recording procedure for maternal behaviour

Starting 10 min after the delivery of the last puppy, behavioural scoring was carried out continuously for the first 15 min of every hour during the first 24 h after whelping. A suitable ethogram based on previous studies^38,46^ was used to record the duration and frequency of mother-puppy interactions (Table 2). Self-directed behaviour was also recorded for the dam during this period, as this might reflect time the mother would invest in herself instead of focusing on the puppies. A total observation time of 6 h was completed for each dam and her litter with two exceptions, a Shih-Tzu and a Cavalier King Charles Spaniel cross Bichon Frise for which only 5 h were analysed due to missing footage caused by technical issues. To control for these variation in observation time and different litter sizes across litters, all variables were divided by the total observation time for each litter and the results, except self-directed behaviour, were subsequently divided by litter size. Thus, this resulted in each litter having an average duration in seconds per hour per puppy for each variable. These values were therefore used in subsequent analysis. All video analysis was conducted using the software BORIS v. 7.9.7^52^.

**Table 2:** Behavioural variables recorded for maternal behaviour^38,46^.

Behaviour	Description	Frequency/duration
Vertical nursing*	Mother nursing at least one puppy while she is in a standing or sitting position	D
Lateral nursing*	Mother nursing at least one puppy while she is in a lateral or dorsal recumbency position	D
Ventral nursing*	Mother nursing at least one puppy while she is in a ventral recumbency position	D
Contact	Mother sitting or lying down while making physical contact with the head or body of at least one puppy	D
Licking and grooming puppies	Mother is licking, grooming or sniffing any of her puppies	D
Self-directed behaviour	Grooming, scratching, licking behaviours directed towards the mother’s own body	D

*Variables were summed together and combined into a single ‘Total nursing’ variable for each litter, which was used in subsequent analysis.

### Statistical analysis

All statistical analyses were carried out using IBM SPSS Statistics (v.26, IBM Corp, Armonk, NY, USA) software with the significance level set at alpha ≤ 0.05. Descriptive statistics (Mean ± SEM) were calculated for parity, litter size, total duration of whelping and average birth interval. With the objective of comparing perinatal mortality with previous studies, the proportions of stillborn puppies, early neonatal mortality (number of dead puppies during the first 72 h postpartum) and perinatal mortality (sum of stillbirths and early neonatal mortality) were calculated for each litter dividing the number of dead puppies by the litter size (mortality at puppy level). The number of litters experiencing at least one stillborn and/or early neonatal mortality was also recorded (mortality at litter level).

#### Constructing the ease of whelping index

The behavioural ease of whelping variables (Table 1) were divided by the total observation time in seconds (pre-parturition period + total whelping duration) for each litter, producing an average duration in seconds or frequency per hour for each dam. An average birth interval was calculated for each litter by dividing the sum of birth intervals for the litter by the litter size minus one. Average durations for sitting and lying down were aggregated together for each subject as these behaviours were considered to be indicative of a relaxed state^53,54^.

To generate the ease of whelping index, a principal component analysis (PCA; correlation matrix, no rotation) was performed on 12 variables including the averaged EoW behavioural variables, total duration of whelping and average birth interval^15^. Kaiser-Meyer-Okin (KMO) measure of sampling adequacy and Bartlett’s sphericity test were checked to ensure that data met the assumptions to conduct the analysis. Principal components with Eigenvalues > 1 were extracted for further analysis. Because the objective of the study was to create an index that captured the difficulty of whelping and assigned a single score to each dam, the extracted principal components were not used as individual variables, but rather employed for constructing an index following the methodology described by Friesen et al.^55^. In summary: first, a principal component weight (PCw) was calculated for each of the selected PCs by dividing each PC’s proportion of total variance by the sum of the variance represented by the five selected PCs. Next, the variable loadings from the unrotated component matrix were multiplied by the corresponding PCw and summed to create variable-specific factor scores. To standardise measurements, the averaged EoW variables values included in the PCA were converted to z-scores using SPSS Statistics 26. Finally, the standardised variable values were multiplied by their corresponding variable-specific factor score and the resulting values were summed to produce a single score for each dam. This method generated an EoW index where a higher score represented higher whelping difficulty, which reflected the periparturient behaviour of the dam.

#### Factors influencing ease of whelping

General Linear Models were used to investigate which factors could have affected whelping difficulty. The EoW index was used as the response variable and parity, litter size (small: < 6 puppies, medium: 6–8 puppies, large: > 8 puppies), origin of dam, breed group and whelping season were used as predictors. A backwards stepwise selection method was used, where predictors were removed one by one, starting with the least significant value, until only significant predictors (Wald’s Chi-squared test, P ≤ 0.05) were left in the model. Akaike information criteria (AIC) values were then compared between models to confirm that the final model was the best fitting one. The performance of the final model versus the intercept was compared using the Likelihood Ratio Chi-Square test and it was considered to be acceptable when the significance level was lower than 0.05. *Post-hoc* comparisons were run using Fisher’s LSD or Tukey HSD tests where appropriate. Results are reported as means with their standard errors (Mean ± SEM).

#### Effect of ease of whelping on maternal behaviour

Following the approach used in previous research^38,46,56^, a PCA was performed on the averaged maternal behaviour variables to summarise the variation into one single component. However, Kaiser–Meyer–Olkin measure of sampling adequacy was too low (KMO = 0.459), indicating that data did not meet the criteria for conducting a PCA^57^. Therefore, the effect of the EoW index on each maternal behaviour variable and on self-directed behaviour was analysed individually using General Linear Models. The three different nursing style variables were aggregated together into a single ‘total nursing’ variable for each litter. Total nursing, licking and grooming puppies, contact, and self-directed behaviour were used as response variables and the EoW index, parity, origin of dam, breed group and whelping season were used as predictors. A backwards stepwise selection method was followed as described above. *Post-hoc* comparisons were run using Fisher’s LSD tests. All results are reported as means with their standard errors (Mean ± SEM).

#### Factors affecting perinatal mortality

To investigate which factors influenced the likelihood of experiencing perinatal mortality, the presence/absence of dead puppies (including stillbirths) during the first 72 h postpartum for each litter was used as the response variable in a binary logistic regression model. The probability distribution used was ‘Binomial’ and the link function was ‘Logit’. The EoW index, parity, litter size, breed group, origin of dam and whelping season were included as predictors. As for previous models, a stepwise selection method was followed and goodness of fit was confirmed comparing AIC values between models. The Likelihood Ratio Chi-Square test was used to compare the performance of the final model versus the intercept and was deemed as adequate with a significance level lower than 0.05.

## Results

### Descriptive statistics

The descriptive statistics for reproductive parameters are presented in Table 3. Of the 224 puppies born, 10 (4.46%) of them were stillborn and 11(4.91%) more died during the first 72 h postpartum. Early neonatal mortality was not observed in any of the litters with stillbirths, thus the perinatal mortality rate was 9.38% (21 puppies). At litter level, perinatal mortality occurred in 12 (40%) of the litters included in this study. At least one stillborn puppy was present in six (20%) of the litters and six (20%) more experienced early neonatal mortality.

**Table 3:** Descriptive statistics for parturition and reproductive parameters of the 30 dams included in the study.

	Mean	SEM	Min	Max
Parity	1.83	0.26	0	4
Litter size	7.47	0.44	3	12
Duration of whelping (min)	369.73	39.86	101.65	877.83
Average birth interval (min)	58.65	6.21	23.03	175.57

### Constructing the ease of whelping index

**Figure 1:**
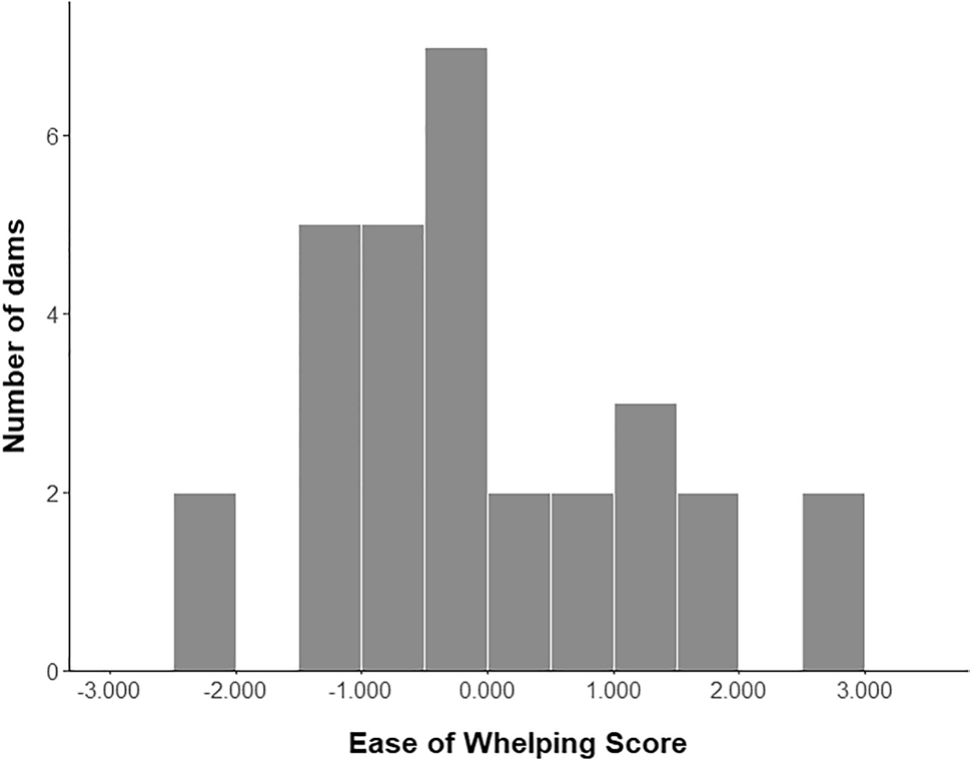
Distribution of the ease of whelping index among the 30 CBE dams included in the study.

The correlation matrix was considered appropriate for performing a PCA^57^ (KMO = 0.579, Bartlett’s sphericity χ^2^ (66) = 217.62, P < 0.001) and subsequently used for principal component extraction. The PCA extracted five components with eigenvalues greater than 1, which together explained 85.44% of the variance. All of the variables had loadings greater than 0.500 in at least one of the extracted PCs, thus they were considered relevant for PC interpretation^57^ and included in the ensuing steps of the analysis. The results of the PCA, including the PC weights and variable-specific factor scores calculated for constructing the EoW index, are presented in Table 4. The analysis produced a standardised ease of whelping index (median = − 0.2359, range = − 2.2486 to 2.9424, Fig. 1) in which an individual score was assigned to each dam. A higher score indicated higher whelping difficulty expressed by the dams as increased activity, restlessness, discomfort and distress, and lower relaxation 24 h before and during parturition.

**Table 4:** Variable loadings, variable-specific factor scores, percentage of variance explained and principal component weights for the principal components (Eigenvalue > 1) extracted by the PCA.

Variable	PC1	PC2	PC3	PC4	PC5	Variable-specific factor score
Sitting and lying down	− 0.800	− 0.298	0.329	− 0.293	0.125	− 0.3017
Standing	0.791	0.074	− 0.388	0.390	0.012	0.2613
Walking	0.736	− 0.147	− 0.014	− 0.225	0.405	0.1395
Position changes	0.699	0.240	− 0.523	− 0.245	− 0.127	0.2149
Vocalisations	− 0.155	0.827	0.361	− 0.125	0.166	0.2922
Circling	0.172	0.686	0.345	− 0.183	0.331	0.3092
Whelping duration	0.435	0.638	0.413	− 0.231	− 0.299	0.0775
Average birth interval	0.568	− 0.283	0.665	0.180	− 0.065	0.2604
Nest building behaviour	0.386	− 0.548	0.599	0.059	− 0.361	0.2282
Self-directed behaviour	− 0.058	0.423	0.242	0.787	− 0.155	− 0.0131
Retching	− 0.432	0.200	− 0.146	0.587	0.207	0.1782
Elimination behaviour	0.373	− 0.377	0.282	0.216	0.635	0.2093
Explained Variance (%)	27.83	20.64	15.94	12.49	8.59	
PC weight (%)	32.57	24.15	18.65	14.62	10.01

### Factors influencing ease of whelping

**Figure 2:**
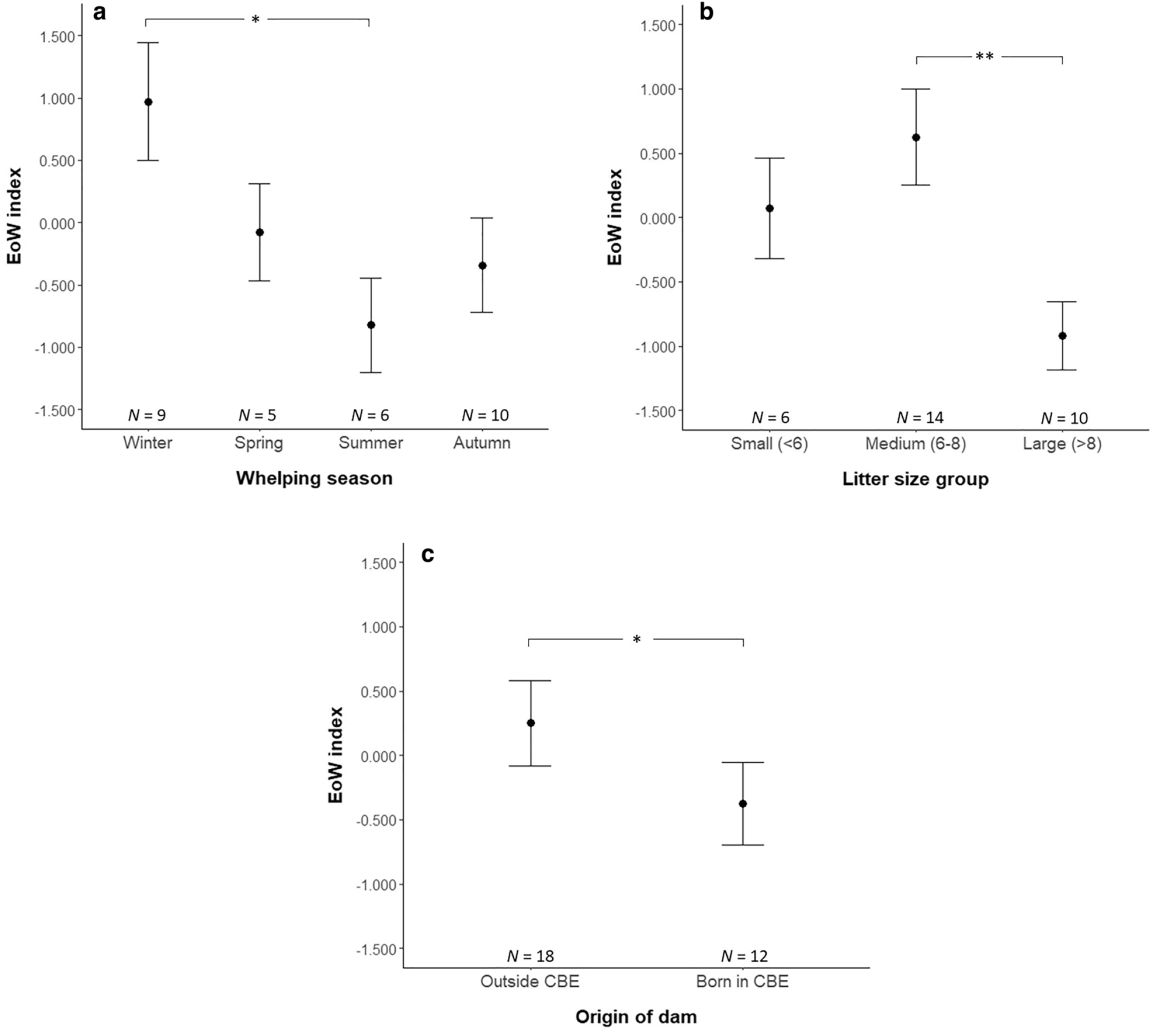
Factors affecting the ease of whelping index on the 30 dams included in the study: whelping season (**a**), litter size group (**b**) and origin of the dam (**c**). Plots show the mean and standard error of the mean for each group of each factor. Stars indicate significant differences between categories (*P < 0.05, **P < 0.01).

Dams ease of whelping score was significantly affected by whelping season (Wald’s χ^2^_(3)_ = 8.194, P = 0.042, Fig. 2a) and litter size group (Wald’s χ^2^_(2)_ = 7.701, P = 0.021, Fig. 2b). A *post-hoc* Tukey HSD test revealed that dams that gave birth during winter (N = 9, 0.970 ± 0.474) had higher EoW scores compared with those giving birth during the summer (N = 6, − 0.822 ± 0.380; P = 0.038). A Fisher’s LSD test showed that dams giving birth to large litters (> 8 puppies: N = 10, − 0.920 ± 0.266) scored lower on average than mothers that whelped medium-sized litters (6–8 puppies: N = 14, 0.625 ± 0.372; P = 0.003). Furthermore, dams whose origin was other than the CBE (N = 18, 0.250 ± 0.333) had higher EoW scores compared with those born and reared within the CBE (N = 12, − 0.375 ± 0.318; Wald’s χ^2^_(1)_ = 4.305, P = 0.038, Fig. 2c). There were no significant effects of parity or breed group on the EoW index.

### Effect of ease of whelping on maternal behaviour

**Figure 3:**
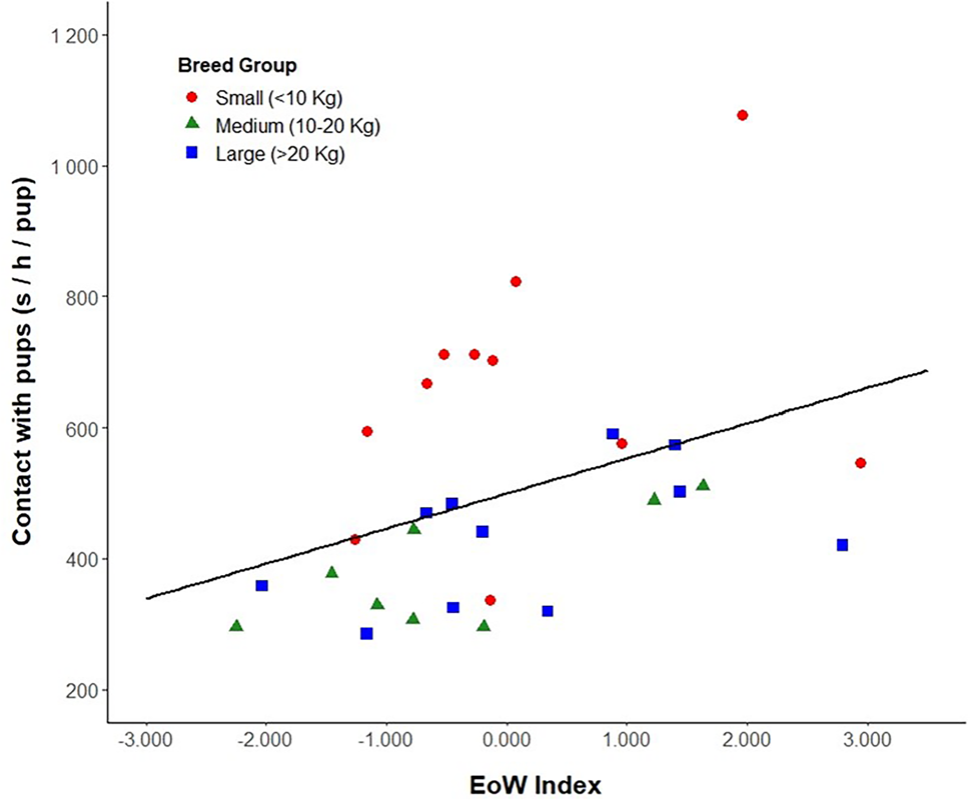
Relationship between the dams ease of whelping index and the average time spent in contact with their puppies during the first 24 h postpartum. The different point shapes represent each dam categorised according to their breed size group. Trend line indicates the line of best fit (P < 0.05).

**Figure 4:**
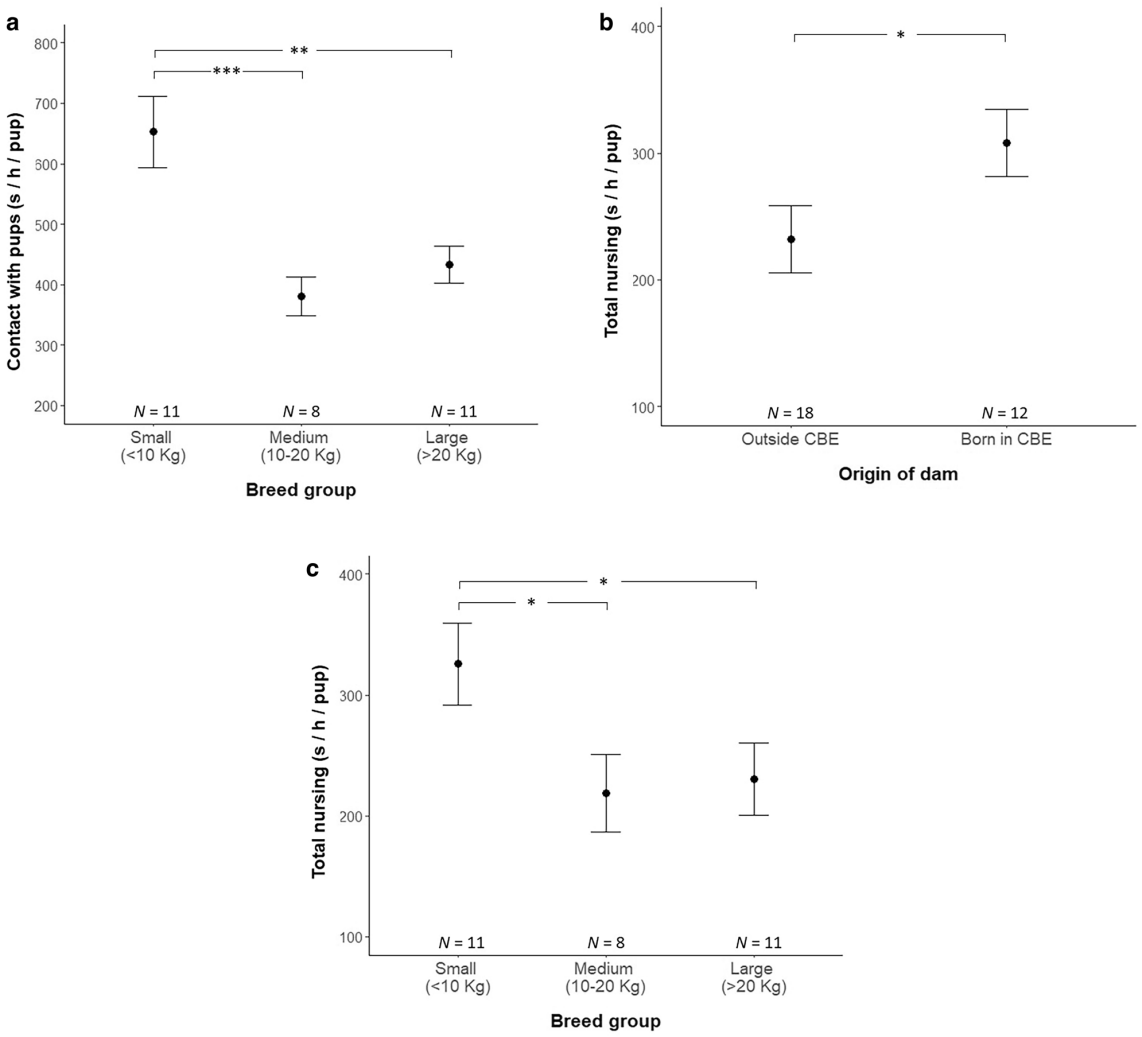
Factors influencing maternal behaviour during the first 24 h postpartum. Time in contact with puppies was affected by breed group (**a**). Total nursing duration was affected by origin of the dam (**b**) and breed group (**c**). Plots show the mean and standard error of the mean for each group of each factor. Stars indicate significant differences between categories (*P < 0.05, **P < 0.01, ***P < 0.001).

From the measured maternal behaviour variables, a significant positive relationship was found between the EoW index and the amount of time mothers spent lying or sitting in contact with their puppies during the first 24 h postpartum (Wald’s χ^2^_(1)_ = 6.037, P = 0.014, Fig. 3). The model showed that contact was also affected by breed group (Wald’s χ^2^_(2)_ = 24.086, P < 0.001, Fig. 4a), with mothers from small breeds (N = 11, 652.597 ± 59.510 s) spending significantly more time on average next to their puppies than mothers from medium-sized (N = 8, 380.632 ± 31.461 s; P < 0.001) and large breeds (N = 11, 433.408 ± 30.898 s; P = 0.001). Contact was not affected by the season of whelping, parity or the origin of the dam.

Total nursing was affected by the origin of the dam (Wald’s χ^2^_(1)_ = 6.254, P = 0.012, Fig. 4b) and breed group (Wald’s χ^2^_(2)_ = 9.905, P = 0.007, Fig. 4c). Mothers born and reared within the CBE (N = 12, 308.224 ± 26.406 s) spent on average more time nursing their puppies than mothers brought into the CBE (N = 18, 231.722 ± 26.516 s). Furthermore, Fisher’s LSD *post-hoc* analyses revealed that mothers from small breeds (N = 11, 325.646 ± 33.648 s) spent on average significantly more time nursing their puppies than mothers from medium-sized (N = 8, 219.080 ± 32.076 s; P = 0.021) and large breeds (N = 11, 230.449 ± 29.685 s; P = 0.025). Self-directed behaviour and the time spent by the mothers licking and grooming their puppies were not significantly affected by the EoW index or any other of the predictors included in the models.

### Factors affecting the likelihood of suffering perinatal mortality

The likelihood of experiencing perinatal mortality was only significantly affected by breed group (Wald’s χ^2^_(2)_ = 8.107, P = 0.017). Litters from medium-sized dogs were significantly less likely (had 0.99 times lower odds) to experience perinatal mortality compared with those born from large breed dogs (Wald’s χ^2^_(1)_ = 6.721, P = 0.010). A non-significant trend was shown for small breed mothers having 0.90 times lower odds of suffering a puppy death compared with large breeds (Wald’s χ^2^_(1)_ = 3.021, P = 0.082). The model also revealed non-significant trends for litter size (Wald’s χ^2^_(1)_ = 3.069, P = 0.080) and EoW index (Wald’s χ^2^_(1)_ = 2.893, P = 0.089). For each 1.0 increase in the EoW index, a non-significant increase of 153.2% was observed in the odds of experiencing perinatal mortality. Similarly, the odds of puppy perinatal mortality were non-significantly increased by 110.1% with each additional puppy in the litter. Results for the binary logistic regression model are presented in Table 5.

**Table 5:** Results of the final binary logistic regression model for factors affecting the likelihood of litters experiencing perinatal mortality (χ^2^_(2)_ = 18.696, P = 0.001, AIC = 31.685).

Predictor	Odds ratio	95% CI	P value
EoW index	2.532	0.868–7.387	0.089
Litter size	2.101	0.916–4.822	0.080
Breed group: large	–	–	Baseline
Breed group: medium	0.010	0.000–0.328	**0.010**
Breed group: small	0.102	0.008–1.338	0.082
Intercept	0.193	–	7.957

Bold indicates P < 0.05.

*CI* confidence interval.

## Discussion

Despite the increasing societal concern about the welfare of dogs maintained in CBEs, it is only recently that a few empirical studies have been conducted on animals living in this environment^58–62^. Dog breeders consider the periparturient period as the most important stage during the breeding process^11^. However, to our knowledge, the factors which affect whelping difficulty in CBE dams and their consequences on early maternal behaviour had not been investigated until now.

The first objective of our study was to create an ease of whelping index based on the behaviour of the dam 24 h before and throughout the whelping process. The PCA used to develop the final EoW index grouped the variables of interest in five PCs where variables with higher positive loadings indicated increased levels of activity, restlessness and distress, longer whelping durations and average birth intervals, and lower levels of relaxation in the periparturient dams (Table 4). Münnich and Küchenmeister^18^ found that a longer duration of the expulsion stage was associated with the occurrence of canine dystocia and the requirement of a caesarean section. Similarly, prolonged parturitions are often considered problematic and associated with dystocia in other domestic species such as pigs and cows^63^. Increased restlessness and discomfort signs (i.e. tail raised for longer) were observed in cows with dystocia when compared with cows that had non-assisted undisturbed calvings^16^ and a higher frequency of postural changes was observed in sows experiencing more difficult farrowings^15^. In the current study, higher durations and frequencies of most ease of whelping behavioural variables (except sitting and lying down) were also interpreted as signs of increased whelping difficulty and expressed as higher scores in the EoW index. Therefore, results suggest that, together with the duration of parturition and birth interval, whelping difficulty can be assessed by observing the behaviour of the dam during the periparturient period. Moreover, the distribution of the EoW scores (Fig. 1) showed that the whelping process can vary in difficulty across dams, although this variation may be influenced by different factors.

In the current study, the EoW index was influenced by litter size, whelping season and the origin of the dam. Regarding the relationship between litter size and the EoW index, results showed that mothers giving birth to nine or more puppies had lower EoW scores in comparison to those with medium-sized litters (6–8 puppies), implying that dams producing larger litters had easier whelpings. In line with this finding, O’Neill et al.^19^ reported that the median total litter size in 425 dystocia cases recorded by veterinary practices in the UK was four (IQR 3–6) puppies. This suggests that difficult births might be more likely to occur in smaller and medium-sized litters rather than in large litters. Wilsson and Sundgren^64^ found that there is a negative relationship between litter size and puppy weight. Therefore, larger puppies from small and medium-sized litters might increase whelping difficulty, particularly in small and medium-sized dogs^65^. A different study found that dogs giving birth to medium-sized litters had a lower risk of experiencing dystocia than those delivering small and large litters^24^. Nevertheless, earlier research showed that litter size was the main cause of dystocia only in 16% of 530 cases, whereas 41.70% of dystocia cases were attributed to maternal causes, mainly due to primary or secondary uterine inertia^18^. Secondary inertia can be a result of a larger litter size increasing the duration of parturition and leading to exhaustion of the myometrium and inhibition of uterine contractions^66^. In our study, whelping duration increased with litter size, thus increasing the dam’s individual EoW score. However, the behaviour of the dam during this period appears to have a bigger weight on her final EoW score, as indicated by each variable’s Factor Score used to calculate the EoW index (Table 4). Although mothers giving birth to larger litters had longer whelpings, they were less active and restless before and during parturition. A possible explanation is that the reduced level of activity may be attributed to exhaustion of the dam due to prolonged whelping. However, if this had been the case, it would be expected for these mothers to spend more time lying next to their puppies following parturition. It is generally accepted that a significant positive relationship exists between the size of the breed and litter size^67–69^. Accordingly, we observed large litters (> 8 puppies) occurring mostly in medium-sized and large breeds. Both breed groups spent less time lying in contact with their litter after delivery of the last puppy. Moreover, the amount of time spent lying in contact with the puppies also increased with whelping difficulty. Therefore, it is likely that the lower levels of activity and restlessness observed in dams that gave birth to larger litters were not caused by physical exhaustion but rather were a reflection of them being more relaxed while experiencing undisturbed and easier whelpings.

To our knowledge, research about the relationship between season and whelping difficulty in dogs is very limited. Two studies found no evidence of caesarean section rate being affected by seasonality^21,65^. Conversely, we found that dams whelping during winter experienced more difficult parturitions compared with those giving birth during the rest of the year, although the difference was only statistically significant when compared to summer. There is no scientific consensus about the effect of seasonality on reproductive performance in dogs. Some studies did not find any influence of season on whelping rate^21,69^, litter size^70–72^ or puppy perinatal mortality^23^. On the other hand, Gavrilovic et al.^73^ reported that whelping rates were higher in winter and spring compared to other seasons. Similarly, increased neonatal mortality has been recorded during the coldest months of the year^22,70^. The relationship between parturition difficulty and seasonality has been more widely studied in cows, with the highest incidence of dystocia cases being recorded during the winter season^74–77^. Along the same lines, the frequency of dystocia appears to be lower in calvings occurring during summer and fall^76,78^. Seasonal variation in the occurrence of dystocia might be closely associated with other factors such as food availability, the dam’s nutritional status during gestation and the offspring’s birth weight^79^. Calving difficulty increases with increasing birth weight^80^ and heavier calves have been reported to be born during autumn and winter compared with warmer seasons^81^. Similarly, exposure to cold stress resulted in ewes giving birth to significantly heavier lambs^82^. Although the biological mechanism behind this phenomenon requires further research, a possible explanation is that under cold weather conditions blood flow is primarily directed towards internal organs (including the uterus) with the aim of maintaining body temperature. This might provide a higher nutrient supply for the foetus, enhancing its growth^79^. Moreover, in some regions cattle are usually housed indoors and receive high energy supplemental feeding during the cooler months of the year, increasing their nutrient intake^83^. The opposite effect has been observed in heat-stressed animals, where blood flow is diverted towards the skin and distal limbs in an effort to dissipate heat^84^, hampering foetal growth due to nutrient deprivation^85,86^. Comparatively, lower individual birth weights have been attributed to reduced nutrient availability during gestation in polytocous species^87^. Czarnecki and Adamski^88^ found that season and litter size significantly affected the birth weight of long-haired guinea pigs. Sows gave birth to larger litters with the lowest average birth weight during spring and female pups born during winter were significantly heavier than those born during summer. The authors attributed the results to nutritional differences across seasons due to lower availability of fresh vegetables and fruits, particularly in winter. In the current study, the average litter size during winter was lower than in summer (6.11 vs 9.16 puppies, respectively). Additionally, the breeder from the CBE where our study was conducted reported dogs normally increased their feed consumption during the colder months. Considering that a significant negative relationship exists between litter size and birth weight in dogs^64^, we can hypothesise that puppies from the winter litters were larger compared to those born from bigger litters during the summer. Therefore, the increased whelping difficulty observed during winter might be more closely related to litter size, rather than seasonality. Nevertheless, several other factors such as age of the bitch, age of the sire, parity, mating method (natural mating or artificial insemination) and breed have also been identified to affect litter size in dogs^68–71,73,89^. Thus, the interaction between these factors and their influence on ease of whelping requires further research.

A notable finding in our study was related to the influence of the origin of the dam on the EoW score and early maternal behaviour. Mothers born and reared within the CBE had significantly easier whelpings and spent more time nursing their puppies during the first 24 h postpartum compared with dams that were born elsewhere. The differences observed in the EoW index between the two groups could be related to the dogs’ ability to cope with the current environment and their stress levels during the periparturient period. Hiby et al.^90^ found that the ability of a dog to adapt to a kennel environment can be influenced by its background (whether previously being a domestic or stray dog) and past experiences in rehoming kennels. The authors reported that owner-relinquished dogs, which presumably had never been housed in a shelter environment, showed an increase in urinary cortisol throughout the first 10 days after being admitted. Oppositely, cortisol levels decreased over the same time period in stray dogs and dogs returning to the kennels after a period of adoption^90^. Furthermore, when entering a kennel environment, the increase in cortisol levels was significantly higher in unhabituated dogs and remained above baseline levels up to 12 weeks after admittance^8^. Similarly, the length of stay in a kennelled environment can influence the behaviour of dogs. Wells et al.^7^ reported that dogs which had been in the shelter for less than six months were more active and alert than dogs that had been captive for longer. Moreover, dogs that had been kennelled for less than a month also spent more time barking^7^. In agreement with these findings, it is possible that in our current study, although dams coming from a different environment had spent at least a few months within the CBE, they still perceived this relatively new environment as stressful. Under this particular CBE management practices, breeding dams are brought into the facility when they are older than six months of age. This means that these dogs are introduced to a new environment after the sensitive socialisation period (3.5 to ~ 12 weeks of age) has ended^91,92^. During this period, negative experiences or insufficient exposure to an ample range of environmental stimuli can reduce the puppy’s capacity to cope with and adapt to novel situations as an adult^93^. As a result, a dog may become highly reactive or develop fear-related and inappropriate avoidance behaviours towards stressful situations^94,95^. Dams born within the CBE had been handled by the breeders and socialised to this environment since birth. Consequently, these dams might have been less stressed and reactive towards their environment during whelping and while nursing their puppies. Bickell et al.^96^ reported that shortly after delivery, calm ewes spent more time on the lambing site and licked their lambs for longer than nervous ewes. We suggest that by being habituated to their current environment, dams born and reared within the CBE were less anxious, active and restless during the periparturient period which resulted in them experiencing easier whelpings and allowing their puppies to suckle for longer.

Apart from the origin of the dam, early maternal behaviour was also affected by the EoW index and breed group. We found a significant positive relationship between whelping difficulty and the amount of time that mothers spent in contact with their puppies throughout the first 24 h postpartum. During the first days postpartum, longer periods of contact between the bitch and her offspring can be beneficial to regulate the puppies temperature and keep them warm^40^. However, the absence of a significant relationship between EoW and other maternal care variables (i.e. nursing and licking and grooming puppies) in our study suggests that maternal behaviour may not necessarily be enhanced by prolonged contact between mother and offspring. In pigs, increasing farrowing difficulty may lead to postpartum exhaustion of the sow, which can affect maternal behaviour and increase piglet mortality^63^. Pain and discomfort reduce the dam’s motivation to nurse and groom her offspring after delivery^48,49,97^. In our study, physical exhaustion may have caused mothers to spend more time resting next to their puppies after experiencing more difficult whelpings. Moreover, increased parturition difficulty is accompanied by higher levels of pain^98,99^. Therefore, it is possible that abdominal pain after whelping may have caused mothers to change position frequently, impeding puppy sucking behaviour.

The influence of breed on maternal behaviour in dogs remains yet to be understood. To our knowledge, only one study has investigated the effect of breed on mother-puppy interactions^46^. The authors compared maternal behaviour scores between three different breeds and found that Labrador Retrievers engaged in maternal behaviour for longer than German Shepherds. Particularly, Labrador Retriever dams spent more time in contact with their puppies than German Shepherd dogs. Other studies have used only one breed^38,100^ or a limited sample size of dogs from different breeds and crossbreeds living in different households^45,56,101^. Therefore, it is difficult to disentangle the confounding effects that factors such as breed, breeding environment, litter size, age and parity can have on maternal behaviour. An advantage of the current study is that although we included dogs from different breeds, all of them were maintained within the same environment under similar management conditions. Additionally, we categorised dams in breed groups according to their breed standard weight/size in an effort to include breed in the analysis. We found that small breed dams had significantly longer durations for average contact with puppies and nursing behaviours than medium-sized and large breeds. Although we controlled for litter size by creating an average duration for each maternal behaviour variable per puppy for each litter, the differences observed between breed groups may be partially explained by the number of puppies in the litter, as a positive relationship exists between breed size and litter size^67^. Accordingly, we observed that small breed dams gave birth mostly to smaller litters (See Supplementary Table S1). Foyer et al.^38^ and Bray et al.^46^ reported that mother-puppy interactions were higher in small litters than in bigger litters. Based on earlier studies in rodents, the authors suggested that larger litters are likely to demand more effort from the mother, causing fatigue and discomfort, and leading her to spend less time with their offspring^102^. It is important to note that these studies analysed maternal behaviour throughout the first three weeks postpartum and our study focused only on the first 24 h as our aim was to investigate if whelping difficulty influenced maternal care during this critical period for the newborn puppy. Interestingly, the positive relationship observed between the EoW index and time in contact with puppies appears to be accentuated in small breed dogs (Fig. 3). Thus, it is possible that the increase in time spent in contact with puppies could be a reflection of longer resting periods due to exhaustion experienced by small breed mothers with smaller litters (and larger puppies) after experiencing more difficult whelpings. We propose that in addition to other factors such as parity^45,46^, breed variation in maternal behaviour expression might be mediated by the effect of litter size on whelping difficulty. However, further research involving a large sample size of breeding dams is needed to clearly understand how these factors interact with each other.

In the current study, the stillbirth rate at puppy level was 4.46%, which is similar to the 4.30% found in a large study conducted in Norway^23^ and lower than the 7% reported by Gill^22^ in a population of purebred dogs in Australia. However, a more recent study found a slightly lower stillbirth rate of 3.26% in a guide dog breeding colony^103^. Similarly, early neonatal mortality in our study (4.91%) was higher than previously reported in other studies. Tønnessen et al.^23^ and Zakošek Pipan et al.^104^ recorded 3.70% and 3.40% puppy deaths respectively during the first week postpartum. Likewise, a survey conducted among French dog breeders reported a puppy mortality rate of 3.40% during the first two days of life^105^. At the litter level, 40% of the litters in our study had at least one dead puppy during whelping and the first 72 h postpartum. This is considerably higher than the 24.60% perinatal mortality reported by Tønnessen et al.^23^ throughout the first week. On the other hand, the proportion of litters in our study with at least one stillborn (20%) was lower than the 28.40% described by Mugnier et al.^105^. Lower perinatal mortality rates observed in previous studies could be explained by a higher monitoring frequency of the litters during whelping and the early neonatal period. Constant supervision of the periparturient dam and throughout the first week postpartum would be expected to occur in professional guide dog breeding kennels^103^ and is likely to facilitate early identification of dystocia and weak puppies^20^. Dos Santos et al.^11^ reported that more than 75% of both professional and occasional dog breeders stayed close to their dogs during parturition to control maternal anxiety after whelping. However, human contact during the periparturient period appears to be significantly reduced in larger facilities, most likely as a result of limited staff^11^. Furthermore, although all whelpings included in our study were considered normal, a few parturitions of dams with higher EoW scores could have been clinically classified as dystocia. The average duration of the expulsion phase is between four and 18 h and puppies are born every 30–120 min without signs of foetal distress^41^. The average birth interval (58.65 min) and average whelping duration (369.73 min) recorded in our study were within these normal ranges. Nevertheless, the longest whelping duration and interbirth interval registered in our study were 14.60 h and 175.57 min respectively. An interbirth interval longer than 2 h and an expulsion phase longer than 12 h are considered within the signs for the occurrence of dystocia^51^, although longer birth intervals up to 4 h can occur without observing evidence of foetal distress or other signs of dystocia^20^. It is possible that due to the high number of dogs maintained in the facility where our study was conducted, breeders were unable to closely supervise all dams during whelping to identify those experiencing more difficult labours and monitor maternal behaviour afterwards.

In our study, the only factor that significantly affected the likelihood of a litter experiencing perinatal mortality was breed size. Medium-sized breeds were significantly less likely to have dead puppies than large breeds. Likewise, a non-significant trend showed that small breed mothers had lower odds of suffering perinatal mortality than large breeds. Our results agree with the literature reporting that perinatal mortality risk increases with breed size, with the highest risk observed in large and giant breeds, both at the puppy and litter level^23^. Larger breeds normally produce bigger litters and thus may have a higher probability of losing at least one puppy^23^. This hypothesis is supported by our results with the trend found for a positive relationship between litter size and the odds of experiencing perinatal mortality. Additionally, prolonged duration of parturition increases the risk of weak and stillborn puppies^18,106^. In our study, dams that had longer whelpings and interbirth intervals were considered to have a higher EoW score. The binomial model for perinatal mortality showed a non-significant trend where dams with higher EoW scores were more likely to have at least one dead puppy. Mugnier et al.^105^ also demonstrated that higher early neonatal mortality in large breeds could be explained by higher within-litter birth weight heterogeneity, which eventually led to the death of lower weight puppies in large litters. Another possibility is that it is easier for a dam to take care of a small litter, reducing the risk of losing a puppy during the first days postpartum. Moreover, puppies from large and giant breeds are at a higher risk of suffering trauma caused by the mother during whelping and in the following days^23^.

Finally, we did not find any significant relationship between the occurrence of perinatal mortality and parity or season of whelping. Primiparous dams have been found at a higher risk of having stillborn puppies but only when they are older than six years of age^18,23^. Although in our study we did not know the exact age of the dams, none of them were older than six years of age and all primiparous mothers were younger than two years old. The risk of experiencing perinatal mortality increases with the age of the mother but decreases with increasing parity^23^. The effect of parity on maternal behaviour is not clear. Guardini et al.^45^ found that maternal behaviour stays consistent in multiparous mothers over the first three weeks postpartum. Furthermore, they show higher levels of maternal care than primiparous mothers during the first week. However, maternal behaviour increases in primiparous mothers during the first 21 days, surpassing multiparous mothers by the end of the third week. On the other hand, a different study found that the expression of maternal behaviour decreased with the more experienced the mother was^46^. Therefore, further research into this subject is required. Regarding the effect of season, only one other study found that a significantly higher number of early neonatal deaths occurred during winter^22^. Given that we observed that mothers giving birth during the winter experienced more difficult parturitions than those giving birth during the rest of the year, we expected to find a similar effect of seasonality in the occurrence of puppy deaths. However, dams in our study were provided with heating lamps days before the expected date of parturition and throughout the first days postpartum. This might have prevented further early neonatal death due to exposure to cold temperatures.

The present study had a few limitations, mainly the fact that it only included subjects from a specific large-scale breeding facility. On the one hand, this reduces environmental variation and enabled us to focus on understanding the relationship between factors such parity, litter size, breed and the periparturient behaviour of the dam. On the other hand, differences between CBEs regarding the environmental conditions of the whelping area, the number of staff, and the management and husbandry practices might also influence the behaviour of the dam during the periparturient period. Additionally, given that behavioural data collection for this study was conducted entirely from video, we were unable to record any physiological measures from either the dams or the puppies. Further research could investigate the relationship between physiological indicators (e.g. dam’s temperature, heart rate variability, salivary cortisol levels, puppies’ mucus colour and heart rate) and the EoW index.

## Conclusion

This is the first study to create an ease of whelping index in dogs based on the quantification of the behaviour of the dam during the periparturient period and to investigate how whelping difficulty, along with other factors, influence early maternal behaviour and puppy perinatal mortality on a large-scale CBE. The behaviour of the dam before and during parturition appears to be a good indicator of whelping difficulty. However, behavioural coding is time-consuming, thus further research could focus on the use of automated devices to measure the behaviour and activity of the dam during this period. The ease of whelping score was affected by whelping season, litter size and the dam’s origin or rearing environment. Maternal behaviour and puppy perinatal mortality were affected by whelping difficulty and breed group, although the effect of these factors appears to be mediated by the size of the litter. Overall, the rate of perinatal mortality in this study was higher than what has been previously reported, particularly at the litter level. These results are relevant for all dog breeders but especially for large scale breeding facilities which may require more staff to adequately monitor a higher number of breeding dams during this critical stage of the breeding cycle. Based on the EoW index, we suggest that in addition to monitoring birth interval and whelping duration, periparturient dams should be closely observed for increased activity, restlessness and distress shown as frequently changing posture, walking around the whelping area and vocalising. The timely identification of breeding dams enduring a difficult whelping process, even without evident dystocia signs, can help breeders to provide increased attention to these mothers and their litters throughout the first days postpartum. Additionally, dams experiencing more difficult whelpings might benefit from postpartum pain management medication.

## Supplementary Information

Supplementary Information.

## Data Availability

The datasets generated and analyzed in this study are available from the corresponding author on reasonable request.
