# Supplementary material for: Factors influencing ease of whelping and its relationship with maternal behaviour and puppy perinatal mortality in commercially bred dogs
Source: Sci Rep. 2022 Apr 23;12:6680. doi: 10.1038/s41598-022-10707-w (PMC9035175; doi:10.1038/s41598-022-10707-w)
Supplement: Supplementary file 1 — Supplementary Information. [file 41598_2022_10707_MOESM1_ESM.docx]

**Factors influencing ease of whelping and its relationship with maternal behaviour and puppy perinatal mortality in commercially bred dogs.**

Uri Baqueiro-Espinosa^1*^, Victoria McEvoy^1^, Gareth Arnott^1^

^1^Institute for Global Food Security, School of Biological Sciences, Queen’s University, Belfast, UK.

**^*^**Corresponding author: ubaqueiroespinosa01@qub.ac.uk

**Supplementary Table S1.** Characteristics of the 30 dams included in the study.

| Breed | Parity | Origin | Whelping season | Litter size |
| --- | --- | --- | --- | --- |
| Samoyed | 0 | Outside CBE | Autumn | 7 |
| Cocker Spaniel | 3 | Outside CBE | Autumn | 10 |
| Labrador Retriever | 2 | Born in CBE | Autumn | 7 |
| Shih-Tzu | 4 | Outside CBE | Autumn | 5 |
| Dachshund | 1 | Outside CBE | Autumn | 6 |
| Cavalier King Charles Spaniel cross Bichon Frise | 4 | Born in CBE | Autumn | 8 |
| Samoyed | 0 | Outside CBE | Autumn | 7 |
| Labrador Retriever | 3 | Born in CBE | Autumn | 12 |
| Cavalier King Charles Spaniel cross Bichon Frise | 3 | Outside CBE | Winter | 3 |
| West Highland Terrier | 3 | Outside CBE | Winter | 6 |
| Cavalier King Charles Spaniel cross Bichon Frise | 3 | Born in CBE | Winter | 6 |
| Samoyed | 0 | Outside CBE | Winter | 6 |
| Cavalier King Charles Spaniel cross Bichon Frise | 3 | Outside CBE | Winter | 5 |
| Samoyed | 0 | Born in CBE | Winter | 7 |
| Labrador Retriever | 0 | Born in CBE | Winter | 9 |
| Cocker Spaniel | 3 | Outside CBE | Winter | 6 |
| Cavalier King Charles Spaniel | 0 | Born in CBE | Winter | 7 |
| Cocker Spaniel | 4 | Outside CBE | Spring | 11 |
| Golden Retriever | 1 | Outside CBE | Spring | 6 |
| Bichon Frise | 3 | Born in CBE | Spring | 5 |
| West Highland Terrier | 2 | Outside CBE | Spring | 9 |
| Cavalier King Charles Spaniel cross Cocker Spaniel | 1 | Born in CBE | Spring | 8 |
| Labrador Retriever | 4 | Outside CBE | Summer | 11 |
| Samoyed | 1 | Outside CBE | Summer | 7 |
| Springer Spaniel | 1 | Outside CBE | Summer | 12 |
| Cocker Spaniel cross French Poodle | 1 | Outside CBE | Summer | 11 |
| Cocker Spaniel | 1 | Born in CBE | Summer | 9 |
| French Poodle | 0 | Born in CBE | Summer | 5 |
| Labrador Retriever | 1 | Born in CBE | Autumn | 9 |
| Bichon Frise | 3 | Outside CBE | Autumn | 4 |
